# Lymph node positivity in different early breast carcinoma phenotypes: a predictive model

**DOI:** 10.1186/s12885-018-5227-3

**Published:** 2019-01-10

**Authors:** Gilles Houvenaeghel, Eric Lambaudie, Jean-Marc Classe, Chafika Mazouni, Sylvia Giard, Monique Cohen, Christelle Faure, Hélène Charitansky, Roman Rouzier, Emile Daraï, Delphine Hudry, Pierre Azuar, Richard Villet, Pierre Gimbergues, Christine Tunon de Lara, Marc Martino, Jean Fraisse, François Dravet, Marie Pierre Chauvet, Jean Marie Boher

**Affiliations:** 1 Institut Paoli Calmettes et CRCM, 232 boulevard de Sainte Marguerite, 13009 Marseille, France; 2 Institut René Gauducheau, Site Hospitalier Nord, St Herblain, France; 3 Institut Gustave Roussy, 114 rue Edouard Vaillant, Villejuif, France; 4 Centre Oscar Lambret, 3 rue Frédéric Combenal, Lille, France; 5 Centre Léon Bérard, 28 rue Laennec, Lyon, France; 6 Centre Claudius Regaud, 20-24 rue du Pont St Pierre, Toulouse, France; 7 Centre René Huguenin, 35 rue Dailly, Saint Cloud, France; 8 Hôpital Tenon, 4 rue de la Chine, Paris, France; 9 Centre Georges François Leclerc, 1 rue du Professeur Marion, Dijon, France; 10 Hôpital de Grasse, Chemin de Clavary, Grasse, France; 11 Hôpital des Diaconnesses, 18 rue du Sergent Bauchat, Paris, France; 12 Centre Jean Perrin, 58 rue Montalembert, Clermont Ferrand, France; 13 Institut Bergonié, 229 Cours de l’Argonne, Bordeaux, France; 14 Aix-Marseille University, Unité Mixte de Recherche S912, Institut de Recherche pour le Développement, 13385 Marseille, France

**Keywords:** Breast cancer, Sentinel node, Risk prediction, Nomogram, Molecular subtype

## Abstract

**Background:**

A strong correlation between breast cancer (BC) molecular subtypes and axillary status has been shown. It would be useful to predict the probability of lymph node (LN) positivity. Objective**:** To develop the performance of multivariable models to predict LN metastases, including nomograms derived from logistic regression with clinical, pathologic variables provided by tumor surgical results or only by biopsy.

**Methods:**

A retrospective cohort was randomly divided into two separate patient sets: a training set and a validation set. In the training set, we used multivariable logistic regression techniques to build different predictive nomograms for the risk of developing LN metastases. The discrimination ability and calibration accuracy of the resulting nomograms were evaluated on the training and validation set.

**Results:**

Consecutive sample of 12,572 early BC patients with sentinel node biopsies and no neoadjuvant therapy. In our predictive macro metastases LN model, the areas under curve (AUC) values were 0.780 and 0.717 respectively for pathologic and pre-operative model, with a good calibration, and results with validation data set were similar: AUC respectively of 0.796 and 0.725.

Among the list of candidate’s regression variables, on the training set we identified age, tumor size, LVI, and molecular subtype as statistically significant factors for predicting the risk of LN metastases.

**Conclusions:**

Several nomograms were reported to predict risk of SLN involvement and NSN involvement. We propose a new calculation model to assess this risk of positive LN with similar performance which could be useful to choose management strategies, to avoid axillary LN staging or to propose ALND for patients with high level probability of major axillary LN involvement but also to propose immediate breast reconstruction when post mastectomy radiotherapy is not required for patients without LN macro metastasis.

**Electronic supplementary material:**

The online version of this article (10.1186/s12885-018-5227-3) contains supplementary material, which is available to authorized users.

## Synopsis

A retrospective cohort of 12.572 early BC patients with SN biopsies was randomly divided into two separate patient sets to develop, validate and compare different predictive nomograms for the risk of developing LN metastases from clinical and pathologic variables provided by tumor surgical results or by biopsy.

## Background

In breast cancer (BC), nodal status is a major prognostic factor that determines therapeutic decisions to a large extent. Sentinel lymph node biopsy (SLNB) provides a reliable assessment of the axilla status in early clinically node-negative BC [1]. Since it also causes less morbidity than axillary lymph node dissection (ALND), it is now considered as a standard of care procedure. The omission of completion ALND in patients with negative sentinel lymph nodes (SLN) has been recognized as a reasonable attitude since the publication of the NSABP B-32 results [2]. Moreover, it is likely that it can be safely expanded to patients with minimal SLN involvement (isolated tumor cells and micro metastases), with regard to survival outcomes [3, 4]. Indeed, 40 to 70% of these patients do not have metastatic non-sentinel lymph nodes (NSLN) [5]. Main predictors of LN metastases are tumor size, grade, lymphovascular invasion (LVI), age at diagnosis, extracapsular extension of the positive SLN, and hormonal and HER2 receptor status [6–10]. In addition, a strong correlation between BC molecular subtypes and /or tumor phenotypes on the one hand (determined by hormonal receptor and HER2 status) and axillary status on the other hand has been shown in numerous studies [11–16].

The determination of the risk of positive axillary LN can significantly contribute to therapeutic decisions. However, this risk cannot be immediately induced from the results of multivariate analyses that provide broad statistical information. Only an appropriate prediction tool, using a nomogram, can indicate the individual risk of a given patient. These nomograms can also be used to compare populations from different studies. A large cohort is necessary to reliably determine the probability of positive SN, particularly for less frequent tumor phenotypes. Reyal et al. published such a nomogram predictive of the risk of developing SN metastases in 2011 [11], built on a training set made of 1543 early-stage BC patients, and validated on two cohorts of 615 and 496 patients respectively. This model was further validated in a cohort of 755 consecutive patients treated at Institut Curie in 2009 [17].

The aim of our study was to develop and compare the performance of multivariable models to predict LN metastases, including nomograms derived from logistic regression with clinical, pathologic variables provided by tumor surgical results or only provided by biopsy as explanatory variables.

## Methods

### Patients

Our cohort consisted of 12,572 consecutive patients with small (≤ 30 mm based on clinical and radiologic findings), clinically node-negative invasive BC, who did not receive neoadjuvant therapy, and underwent SLNB between 1999 and 2012 at 13 French centers. HER2 status was determined for all patients. During the first years of the study, ALND was systematically performed in some sites; thereafter, ALND was performed only in case of SN involvement, this attitude being homogeneous within all the participating sites.

### Evaluation

The following data were retrieved: characteristics of patients (age at the time of SLNB), and tumors [size, clinical stage, histological type, estrogen (ER), progesterone (PR) and HER2 status, LVI, Scarff-Bloom-Richardson (SBR) grade], description of ALND (number of LN sampled and involved), and results of the pathological examination of surgical resection specimens. Tumor size was determined on the results of pathological examination but could be evaluated pre operatively by mammography, sonography and in selected cases by MRI (clinical T stage). LVI was detected on surgical specimen.

Tumor phenotype was defined by the combination of ER, PR and HER2 status, evaluated by immuno-histochemistry (IHC) and confirmed by FISH in case of IHC-HER2 2+. Positivity for ER and PR was determined according to French guidelines (≥ 10% of cancer cells expressing ER/PR). Five molecular subtypes were defined according to clinico-pathological criteria [18]. Because information on Ki-67 was not available, we used grade to capture cell proliferation, as described by von Minckwitz et al [19] The following definitions were used: triple-negative (basal-like, HER2-/HR-), HER2 positive (non-luminal, HER2+/HR-), and luminal (HR+), divided into luminal A (HR+/HER2−/grade1 or 2), luminal B-HER2-negative like (HR+/HER2−/grade 3), and luminal B-HER2-positive like (HR+/HER2+ all grades).

Although the methods used for histological examination were not standardized in the protocol, all sites proceeded similarly: serial sections were performed every 200 μm and stained with standard hematoxylin and eosin. The number of sections was six to ten, or pursued until node exhaustion in case of large SN. Additional IHC analysis was done in case of negative results at standard examination. For additional nodes identified by completion ALND, routine HE analysis was performed.

Five categories of LN status were defined: negative LN (pN0i-), isolated tumor cells (pN0(i+): < 0.2 mm), detected either by hematoxylin and eosin (HE) staining or by cytokeratin IHC, micro metastases (pN1mi: > 0.2 mm and < 2 mm), and macro metastases (> 2 mm), divided into single and multiple macro metastases [20].

## Statistical methods

Our main objective was to create prediction models for the risk of LN positivity and the risk of LN macroscopic metastases from clinical and pathologic variables provided by tumor surgical results or by biopsy, and evaluate their performance with respect to three main features: discrimination (i.e. whether the relative ranking of individual predictions is in the correct order)*, calibration* (i.e. agreement between observed outcomes and predictions) *and clinical utility defined as* proportions of patients classified into risk categories using predefined cutoff values (< 10%, between 10 and 20%, between 20 and 30%, between 30 and 40%, and > = 40%). Our main evaluation criteria were based on the final status of LN metastases (pN0(i+), pN1mi or pN1ma) as the result of SLNB alone or the final result of both SLNB and ALND. LN positivity was defined as the presence of isolated tumor cells, micro or macro LN metastases. We used logistic regression models [21] including age (<=40, 41–75,> 75), tumor size (<=20, 20–30, > = 30 mm) or clinical T stage (T0-T1, T2, T3-T4), tumor grade, histology type, LVI, and molecular subtypes as predictor factors to predict each individual risks. The list of predictor factors was set beforehand, based on the investigator’s experience and some reference papers [6–11, 13–15, 17]. No additional procedure was used in regression analysis to reduce the list of only 5 or 6 predictor factors identified beforehand. Prior to analysis, we randomly divided our initial cohort (*N* = 12,572) in two separate sub-cohorts: a large training cohort (*N* = 8381) to create prediction models and a confirmatory cohort (*N* = 4191) to evaluate their individual’s prediction performance. A split-sample approach was adopted in order to estimate unbiasedly the model performance, as these estimates are known to be biased upwards when regression parameters are estimated on the same dataset [22]. First we performed a descriptive analysis using the following criteria: patient’s age at SN biopsy, clinical and pathological tumor size, tumor grade and histology type, lymphovascular invasion or not (LVI), presence of estrogen (ER), progesterone (PR) and hormonal receptors (RH), Her2 positivity, tumor subtype, number of SN removed and final LN status. The evaluation of each model was assessed in the training sample and the confirmatory sample. Differences in patient’s and tumor’s characteristics were compared using Chi Square or exact Fisher test, Student or Wilcoxon rank sum tests as appropriate. The discrimination ability was evaluated by the area under the ROC (Receiver Operating Characteristic) curve (AUC). We used the functions roc and pROC implemented in R to estimate AUC with 95%CI and test for difference in AUCs along the Delong’s method in the confirmatory sample [23]. Empirical distributions of AUC observed after re-fitting a model on bootstrap replicates (B = 2000) were used to estimate AUC and difference in AUCs with 95% Ci in the training sample. Model calibration was evaluated using Hosmer goodness-of-fit test [24]. All statistical analyses were conducted in the R Language and Environment for Statistical Computing version 3.2.5 (The R Foundation, Vienna, Austria).

## Results

### Patients’ characteristics

Patients’ main characteristics are summarized in Table 1. SBR grade was 1, 2 and 3 in 34, 46 and 20% of cases respectively. Hormone receptor-positive tumors (ER+ and/or PR+) accounted for 88% of cases (11,013 patients). Final LN status, taking into account ALND results when performed, was: pN0(i-) in 8253 patients (66%), pN0(i+) in 355 (3%), pN1mi in 970 (8%) and macro metastasis in 2994 (24%). The comparison between patients with positive and negative final LN status, and between patients with LN macro metastases versus pN0 or pNo(i+) or pN1mi showed statistically significant differences with regard to age, pathologic tumor size, SBR grade, LVI, histological type and distribution of molecular subtypes (Tables 2 and 3).

**Table 1:** Population: all patients and patients according to initial data set or validation set

	All patients		Initial set		Validation set	
	Nb	%	Nb	%	Nb	%
Nb patients	12,572		8381		4191	
Age median (range)	58 (18–101)		58 (18–101)		58 (18–100)	
< 60	7231	58	4857	58	2374	57
61–65	1810	14	1166	14	644	15
> 65	3525	28	2355	28	1170	28
Median tumor size	14		14		14	
< 10	4701	38	3136	38	1565	38
11 to 20	5053	41	3368	41	1685	41
> 20	2679	22	1784	22	895	22
No SN removed						
1	3268	31	2203	31	1065	31
2	3374	32	2231	31	1143	33
3	2034	19	1382	20	652	19
> 4	1886	18	1271	18	615	18
Tumor type						
Ductal	9793	78	6522	78	3271	78
Lobular	1645	13	1110	13	535	13
Mixt	226	2	144	2	82	2
Others	899	7	599	7	300	7
Grade						
1	4246	34	2891	35	1355	33
2	5756	46	3800	46	1956	47
3	2448	20	1611	19	837	20
LVI						
Negative	8430	78	5661	78	2769	77
Positive	2400	22	1595	22	805	23
Estrogen receptors						
negative	1730	14	1146	14	584	14
positive	10,828	86	7227	86	3601	86
Progesterone receptors						
negative	3464	29	2281	29	1183	30
positive	8522	71	5701	71	2821	70
Hormonal receptors						
negative	1541	12	1020	12	521	12
positive	11,013	88	7349	88	3664	88
Her2 status						
negative	11,350	90	7570	90	3780	90
positive	1222	10	811	10	411	10
Tumor sub types						
Luminal A	8998	72	6026	72	2972	71
Luminal B Her2-	1178	9	756	9	422	10
HR+ Her2+	766	6	521	6	245	6
HR- Her 2+	450	4	288	3	162	4
Triple Negative	1091	9	732	9	359	9
pN final						
pN0(i-)	8253	66	5507	66	2746	66
pN0(i+)	355	3	233	3	122	3
pN1mi	970	8	660	8	310	7
Macro	2994	24	1981	24	1013	24
Clinical size						
T0	2764	23	1831	23	933	23
T1	6784	57	4544	57	2246	56
T2	2115	18	1383	17	732	18
> T3	283	2	187	2	96	2

**Table 2:** Initial data set and validation set results according to axillary nodal involvement

	Initial set					Validation set				
	pN0		i+/mi/macro			pN0		i+/mi/macro		
	Nb	%	Nb	%	p	Nb	%	Nb	%	*p*
Nb patients	5507		2874			2746		1445		
Age median (range)	59 (18–101)		55 (20–98)			59 (18–100)		56 (22–90)		
< 60	3001	55	1856	65	< 0.0001	1464	53	910	63	< 0.0001
61–65	818	15	348	12		443	16	201	14	
> 65	1686	31	669	23		836	30	334	23	
Tumor size (median)	12		19			12		20		
< 10	2642	49	494	17	< 0.0001	1340	49	225	16	< 0.0001
11 to 20	2185	40	1183	41		1081	40	604	42	
> 20	608	11	1176	41		294	11	601	42	
Tumor type										
Ductal	4244	77	2278	79	< 0.0001	2133	78	1138	79	< 0.0001
Lobular	711	13	399	14		332	12	203	14	
Mixt	73	1	71	2		38	1	44	3	
Others	473	9	126	4		241	9	59	4	
Grade										
1	2142	39	749	26	< 0.0001	1007	37	348	24	< 0.0001
2	2410	44	1390	49		1245	46	711	49	
3	887	16	724	25		459	17	378	26	
LVI										
Negative	4077	89	1584	59	< 0.0001	2004	89	765	58	< 0.0001
Positive	510	11	1085	41		256	11	549	42	
Estrogen receptors										
negative	747	14	399	14	0.6964	371	14	213	15	0.296
positive	4757	86	2470	86		2371	86	1230	85	
Progesterone receptors										
negative	1568	30	713	26	0.0004	782	30	401	29	0.6346
positive	3678	70	2023	74		1841	70	980	71	
Hormonal receptors										
negative	660	12	360	13	0.4835	337	12	184	13	0.6948
positive	4841	88	2508	87		2406	88	1258	87	
Her2 status										
negative	5837	91	1733	87	< 0.0001	2909	92	871	86	< 0.0001
positive	563	9	248	13		269	8	142	14	
Tumor sub types										
Luminal A	4100	75	1926	67	< 0.0001	2029	75	943	66	< 0.0001
Luminal B Her2-	364	7	392	14		217	8	205	14	
HR+ Her2+	336	6	185	6		140	5	105	7	
HR- Her 2+	151	3	137	5		93	3	69	5	
Triple Negative	509	9	223	8		244	9	115	8	
Clinical size										
T0	1458	28	373	14	< 0.0001	756	29	177	13	< 0.0001
T1	3166	61	1378	50		1565	60	675	48	
T2	546	11	837	30		272	10	460	33	
> T3	18	0	169	6		16	1	80	5

**Table 3:** Initial data set and validation set results according to axillary nodal macro metastasis involvement

	Initial set					Validation set				
	pN0 pN1mi		macro			pN0pN1mi		macro		
	Nb	%	Nb	%	p	Nb	%	Nb	%	p
Nb patients	6400		1981			3178		1013		
Age median (range)	58.4 (18–101)		55 (20–98)			59 (18–100)		56 (25–90)		
< 60	3573	56	128/4	65	< 0.0001	1743	55	631	62	0.0002
61–65	934	15	232	12		510	16	134	13	
> 65	1891	30	464	23		922	29	248	24	
Tumor size										
< 10	2872	45	264	13	< 0.0001	1453	46	112	11	< 0.0001
11 to 20	2665	42	703	36		1310	42	375	37	
> 20	786	12	998	51		380	12	515	51	
Tumor type										
Ductal	4981	78	1541	78	< 0.0001	2494	79	777	77	< 0.0001
Lobular	800	13	310	16		382	12	153	15	
Mixt	82	1	62	3		43	1	39	4	
Others	531	8	68	3		257	8	43	4	
Grade										
1	2442	39	449	23	< 0.0001	1147	37	208	21	< 0.0001
2	2841	45	959	49		1453	46	503	50	
3	1048	17	563	29		540	17	297	29	
LVI										
Negative	4655	86	1006	55	< 0.0001	2287	86	482	53	< 0.0001
Positive	780	14	815	45		373	14	432	47	
Estrogen receptors										
negative	827	13	319	16	0.0003	414	13	170	17	0.0031
positive	5570	87	1657	84		2760	87	841	83	
Progesterone receptors										
negative	1751	29	530	28	0.4585	885	29	298	30	0.6346
positive	4330	71	1371	72		2140	71	681	70	
Hormonal receptors										
negative	732	11	288	15	0.0002	375	12	146	14	0.0306
positive	5662	89	1687	85		2800	88	864	86	
Tumor sub types										
Luminal A	4780	75	1246	63	< 0.0001	2345	74	627	62	< 0.0001
Luminal B Her2-	458	7	298	15		272	9	150	15	
HR+ Her2+	383	6	138	7		161	5	84	8	
HR- Her 2+	178	3	110	6		106	3	56	6	
Triple Negative	554	9	178	9		269	9	90	9	
Clinical size										
T0	1601	26	230	12	< 0.0001	828	27	105	11	< 0.0001
T1	3731	61	813	43		1835	61	405	42	
T2	710	12	673	36		348	11	384	40	
> T3	30	0	157	8		20	1	76	8	

**Fig. 1:**
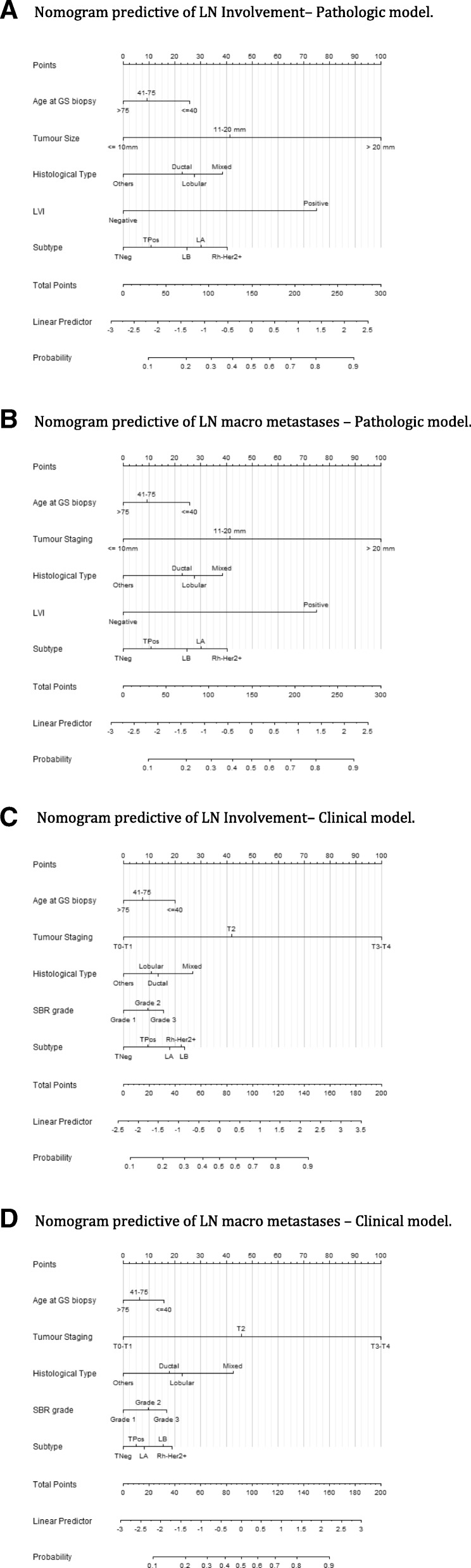
Nomograms. 1a: Nomogram predictive of LN Involvement– Pathologic model. 1b: Nomogram predictive of LN macro metastases – Pathologic model. 1c: Nomogram predictive of LN Involvement– Clinical model. 1d: Nomogram predictive of LN macro metastases – Clinical model.

We first predicted the individual probabilities of final LN positivity and of detecting LN macro metastases from selected clinico-pathologic predictor factors provided by tumor surgical results. The model AUCs with 95% CIs for confirmatory and training samples were respectively 0.767 [0.750–0.783] and 0.755 [0.744–0.767]. Calibration plot and Hosmer-Lemeshow test revealed that the calibration is adequate (*p* = 0.332 in confirmatory sample, *p* = 0.158 in training sample). With respect to clinical utility in confirmatory and training samples, the probability of positive LN were respectively below 10% for 7 patients (< 1%) and 19 patients (< 1%), between 10 and 20% for 1096 (31%) and 2255 (32%), and ≥ 20% for 2409 (68.6%) and 4859 (68.1%) patients (Table 4) (Fig. 1A, Additional file 1: Figure S1A and Additional file 2: Figure S2A). The second pathological model estimated the probability of detecting LN macro metastases only. The AUC values for confirmatory and training samples were respectively 0.798 (0.780–0.815) and 0.780 [0.767–0.790]. Clinical utility measures, estimated the probability of LN macro metastases respectively in confirmatory and training samples below 10% for 1004 patients (29%) and 2029 patients (28%), between 10 and 20% for 1075 patients (31%) and 2289 patients (32%), and >  20% for 1433 patients (41%) and 2815 patients (39.4%). The Hosmer-Lemeshow test revealed a poor calibration of the model (*p* = 0.024 in confirmatory sample, *p* = 0.427 in training sample) (Table 4 and Additional file 1: Table S1) (Fig. 1B, Additional file 2: Figure S1B, Additional file 3: Figure S2B).

**Table 4:** Discrimination, calibration and clinical utility measures of pathologic and pre-operative prediction models

		Pathologic model		Pre-operative model	
Probability of LN positivity					
Criteria	Parametre	Initial set	Validation set	Initial set	Validation set
AUC	Est	0.754	0.767	0.681	0.687
	95%CI	[0.742–0.765]	[0.75–0.783]	[0.668–0.693]	[0.669–0.705]
AUC (Bootstrap,	Est	0.755	0.766	0.682	0.686
B = 2000)	95%CI	[0.744–0.767]	[0.762–0.769]	[0.669–0.694]	[0.682–0.69]
Clinical utility	< 10%	19 (0%)	7 (0%)	1 (0%)	0 (0%)
	10–20%	2255 (32%)	1096 (31%)	579 (7%)	279 (7%)
	20–30%	1258 (18%)	586 (17%)	3137 (40%)	1487 (38%)
	30–40%	1108 (16%)	559 (16%)	2478 (32%)	1315 (33%)
	40–50	391 (5%)	188 (5%)	228 (3%)	125 (3%)
	> = 50%	2102 (29%)	1076 (31%)	1429 (18%)	746 (19%)
Calibration	*p*-value	0.158	0.332	0.815	0.200
Probability of macrometastases					
AUC (Delong)	Est	0.780	0.798	0.718	0.727
	95%CI	[0.767–0.792]	[0.78–0.815]	[0.703–0.732]	[0.707–0.746]
AUC (Bootstrap,	Est	0.780	0.796	0.717	0.725
B = 2000)	95%CI	[0.767–0.793]	[0.793–0.799]	[0.703–0.732]	[0.721–0.728]
Clinical utility	< 10%	2029 (28%)	1004 (29%)	358 (5%)	184 (5%)
	10–20%	2289 (32%)	1075 (31%)	5049 (64%)	2450 (62%)
	20–30%	512 (7%)	262 (7%)	829 (11%)	465 (12%)
	30–40%	726 (10%)	358 (10%)	307 (4%)	162 (4%)
	40–50	644 (9%)	378 (11%)	573 (7%)	306 (8%)
	> = 50%	933 (13%)	435 (12%)	736 (9%)	385 (10%)
Calibration	p-value	0.427	0.024	0.568	0.174

We evaluated the loss in discrimination ability in pre-operative prediction models omitting the information about LVI and substituting pathological tumor size information by clinical T stage. For the overall probability of LN positivity, the AUC values for confirmatory and training samples were respectively 0.687 [0.669–0.705] and 0.682 (0.669–0.694). For the probability of detecting LN macro metastases, the observed AUC results for confirmatory and training samples were respectively 0.727 [0.707–0.746] and 0.717 (0.703–0.732). The calibration of both pre-operative models was found satisfactory. (Table 4) (Fig. 1 C-D, Additional file 2: Figure S1C-D, Additional file 3: Figure S2C-D).

The change in AUCs between pathological and per-operative model were found statistically significantly decreased (*p* < 0.001). We also evaluated in the confirmatory sample the discrimination ability of the prediction models obtained when treating the variable age and tumor size as continuous. The AUC values for predicting LN positivity and the presence of LN metastases were respectively 0.774 [0.758, 0.79] and 0.805 [0.789–0.823]. The observed increases were significantly (*p* = 0.041 and *p* = 0.026), but the results in terms of calibration were judged inadequate (Hosmer-Lemeshow *p* value < 0.001).

## Discussion

The aim of this study was to better understand the relationships between tumor characteristics and the probability of axillary LN positivity. The large cohort used in our study is appropriate for less frequent tumor phenotypes (namely Her2+ and HR-Her2-). We distinguished between various histological tumor types, showing a lower LN positivity rate in tumors other than ductal, lobular or mixt, as previously reported for BC with favorable histology (tubular, mucinous, papillary, medullary, adenoid cystic and secretory) that are associated with a very low LN positivity rate [25].

In our model, we used the same independent variables as Reyal et al. [11], namely age, tumor size, molecular subtypes and LVI, and we added grade and histological type. However, age intervals were different, as well as tumor phenotype definitions (ER only in the Reyal model) and tumor size description (continuous variable in the Reyal model). We obtained different odds ratios for the same variables and clinical utility results were different and higher for low probability of positive lymph node, particularly for macro metastases in our population for both models. Clinical utility results for low probability of positive lymph node could be contributive to avoid surgical axillary staging by sentinel lymph node biopsy or axillary lymph node dissection.

The models were less reliable when information about LVI was missing. LVI could be detected on pre-operative biopsies but the difference in accuracy is obviously large in comparison with surgical specimen analysis.

The HER2 status was unknown in old studies [8] and others studies were based on small number of patients. We found that HER2 negative tumors were associated with LN positivity less frequently than HER2 positive tumors (22.9% vs. 31.9%). Lu et al. published that the lowest probability of node metastasis was for ER- / HER2- tumors [12]. Similarly in our study, triple negative tumors had the lowest probability of node metastasis, while HR- / Her2+ tumors had the highest probability. Reyal et al. hypothesized that the axillary LN metastatic process is predominantly related to intrinsic biological properties in ER-negative and HER2-negative BC, while tumor size, proliferation rate and LVI are the main determinants in the ER positive or HER2 positive breast cancers. However, positive axillary lymph nodes in triple negative BC were pejorative prognostic factors for sentinel node macro-metastases but also for occult sentinel node involvement (pN0(i+) and pN1mi) [26].

A reliable predictive model of LN positivity, based on pathologic parameters, can be used to compare populations from different studies, particularly for trials with or without axillary surgical procedure. Above all, it might allow avoiding SN biopsy when the probability of positivity is very low (< 10%). Some authors already suggested that SN biopsy could be omitted in tumors with good-prognosis subtypes [25] or that axillary dissection is useless in older patients [27]. We believe that these criteria lack accuracy and we prefer a decision-making approach, based on molecular subtypes. However, we must be aware of the risk of insufficient treatment in small tumors with favorable prognostic factors, in which LN status is a major determinant of adjuvant chemotherapy and regional radiotherapy. Moreover, the model is less reliable when LVI is not documented, which is usually the case before surgery. Ultra-sonography of the axilla and percutaneous biopsy is a growing practice. These clinical predictive tools may be helpful relative to the use of axillary ultra-sonography with percutaneous LN biopsy for patients with high level risk of axillary LN involvement.

These models can also be contributive in order to determined indications of post mastectomy radiotherapy for patients with axillary lymph nodes macro-metastases [28], particularly when immediate breast reconstruction can be proposed.

## Conclusions

We reported a reliable predictive model of LN positivity according to different early breast carcinoma phenotypes in a large cohort. The determination of the risk of positive axillary LN can significantly contribute to therapeutic decisions. These models, with or without LVI results, can also be used to determine the risk of positive axillary LN or the risk of LN macro-metastasis. Before surgery, clinical models can be used to propose SLNB or not according to LN involvement probability. After surgery, in case of SLNB omission, if LN involvement probability is high, with eventually modifications of adjuvant treatment indications according to LN status, a re-operation can be proposed (SLNB or cALND). Thus clinical and pathologic models should be helpful in surgical planning, in the setting of a clinical trial and in clinical practice to avoid SLNB for very low risk of LN involvement and to avoid re-operation in case of SLNB omission or to propose ALND for patients with high level probability of major axillary LN involvement but also to propose immediate breast reconstruction when PMRT is not required for.

## Additional files

Additional file 1: **Table S1.** Logistic regression results. (DOCX 22 kb)

Additional file 2: **Figure S1.** Calibration plots of our models. 1A: Calibration of predictive LN Involvement for validation set– Pathologic model. 1B: Calibration of predictive LN macro metastases for validation set – Pathologic model. 1C: Calibration of predictive LN Involvement for validation set – Clinical model. 1D: Calibration of predictive LN macro metastases for validation set – Clinical model. (DOCX 45 kb)

Additional file 3: **Figure S2.** ROC curves of our models. 2A: ROC curves of predictive LN Involvement – Pathologic model. 2B: ROC curves of predictive LN macro metastases – Pathologic model. 2C: ROC curves of predictive LN Involvement – Clinical model. 2D: ROC curves of predictive LN macro metastases – Clinical model. (DOCX 44 kb)
