# Supplementary material for: Lymph node positivity in different early breast carcinoma phenotypes: a predictive model
Source: BMC Cancer. 2019 Jan 10;19:45. doi: 10.1186/s12885-018-5227-3 (PMC6327612; doi:10.1186/s12885-018-5227-3)
Supplement: Supplementary file 1 — Table S1. Logistic regression results. (DOCX 22 kb) [file 12885_2018_5227_MOESM1_ESM.docx]

*Additional file 1****: Table S1*** *Logistic regression results.*

| **Logistic regression results according to axillary nodal involvement** | | | | | |
| --- | --- | --- | --- | --- | --- |
| **Pathologic model** | **Training set** | | | | |
| **Param** | **Coef** | **OR** | **Lower** | **Upper** | **P-value** |
| Intercept | -1.31 | 0.27 | 0.211 | 0.345 | < 0.001 |
| age 41-75 years | -0.302 | 0.739 | 0.595 | 0.919 | 0.00648 |
| age >75 years | -0.472 | 0.624 | 0.465 | 0.837 | 0.00162 |
| Tumor size 11-20 mm | 0.756 | 2.13 | 1.864 | 2.433 | < 0.001 |
| Tumor size > 20 mm | 1.836 | 6.271 | 5.355 | 7.344 | < 0.001 |
| Lobular | 0.074 | 1.077 | 0.914 | 1.268 | 0.37559 |
| Mixt | 0.277 | 1.319 | 0.864 | 2.014 | 0.19955 |
| Others histology | -0.407 | 0.666 | 0.527 | 0.841 | < 0.001 |
| Grade 2 | 0.048 | 1.049 | 0.922 | 1.194 | 0.46661 |
| Grade 3 | -0.147 | 0.863 | 0.645 | 1.155 | 0.32293 |
| LVI : yes | 1.372 | 3.943 | 3.449 | 4.508 | < 0.001 |
| Luminal B | 0.073 | 1.076 | 0.778 | 1.487 | 0.65844 |
| HR+ Her2+ | -0.284 | 0.753 | 0.583 | 0.973 | 0.02982 |
| Triple Negative | -0.424 | 0.654 | 0.496 | 0.864 | 0.00280 |
| HR- Her2+ | 0.296 | 1.344 | 0.956 | 1.89 | 0.08843 |
| **Pre operative model** | **Training Set** | | | | |
| Intercept | -0.731 | 0.481 | 0.39 | 0.595 | < 0.001 |
| agecd=41-75 | -0.399 | 0.671 | 0.55 | 0.818 | < 0.001 |
| agecd=>75 | -0.638 | 0.528 | 0.4 | 0.697 | < 0.001 |
| stade_tclinique=T2 | 1.331 | 3.785 | 3.339 | 4.291 | < 0.001 |
| stade_tclinique=T3-T4 | 3.184 | 24.143 | 14.55 | 40.061 | < 0.001 |
| typehisto_t=Lobulaire | -0.082 | 0.921 | 0.793 | 1.07 | 0.28350 |
| typehisto_t=Mixte | 0.433 | 1.542 | 1.062 | 2.239 | 0.02283 |
| typehisto_t=Autres | -0.425 | 0.654 | 0.525 | 0.814 | < 0.001 |
| grade_sbr=Grade 2 | 0.306 | 1.358 | 1.207 | 1.528 | < 0.001 |
| grade_sbr=Grade 3 | 0.494 | 1.639 | 1.258 | 2.135 | < 0.001 |
| ss_type2=Luminaux B | 0.185 | 1.203 | 0.893 | 1.621 | 0.22330 |
| ss_type2=Rh+Her2+ | -0.268 | 0.765 | 0.604 | 0.969 | 0.02609 |
| ss_type2=Triple Neg | -0.571 | 0.565 | 0.438 | 0.729 | < 0.001 |
| ss_type2=Rh-Her2+ | 0.142 | 1.153 | 0.849 | 1.566 | 0.36346 |
| **Results according to**  **Pathologic model** | **axillary nodal macro-metastasis**  **Training set** | | | | |
| **Param** | **Coef** | **OR** | **Lower** | **Upper** | **P-value** |
| Intercept | -2.227 | 0.108 | 0.082 | 0.142 | < 0.001 |
| age 41-75 years | -0.214 | 0.807 | 0.64 | 1.019 | 0.07113 |
| age >75 years | -0.326 | 0.722 | 0.526 | 0.991 | 0.04376 |
| Tumor size 11-20 mm | 0.709 | 2.032 | 1.719 | 2.402 | < 0.001 |
| Tumor size > 20 mm | 2.107 | 8.224 | 6.879 | 9.831 | < 0.001 |
| Lobular | 0.297 | 1.346 | 1.125 | 1.61 | 0.00115 |
| Mixt | 0.563 | 1.756 | 1.134 | 2.718 | 0.01155 |
| Others histology | -0.553 | 0.575 | 0.429 | 0.772 | < 0.001 |
| Grade 2 | 0.072 | 1.075 | 0.925 | 1.249 | 0.34798 |
| Grade 3 | -0.034 | 0.967 | 0.704 | 1.327 | 0.83343 |
| LVI : yes | 1.184 | 3.267 | 2.846 | 3.751 | < 0.001 |
| Luminal B | 0.117 | 1.124 | 0.796 | 1.587 | 0.50583 |
| HR+ Her2+ | -0.085 | 0.919 | 0.693 | 1.217 | 0.55336 |
| Triple Negative | -0.106 | 0.899 | 0.665 | 1.216 | 0.49086 |
| HR- Her2+ | 0.475 | 1.608 | 1.124 | 2.301 | 0.00939 |
| **Pre operative model** | **Training set** | | | | |
| Intercept | -1.587 | 0.205 | 0.162 | 0.259 | < 0.001 |
| agecd=41-75 | -0.299 | 0.742 | 0.598 | 0.92 | 0.00665 |
| agecd=>75 | -0.507 | 0.602 | 0.443 | 0.818 | 0.00119 |
| stade_tclinique=T2 | 1.47 | 4.349 | 3.823 | 4.948 | < 0.001 |
| stade_tclinique=T3-T4 | 3.21 | 24.779 | 16.403 | 37.431 | < 0.001 |
| typehisto_t=Lobulaire | 0.159 | 1.172 | 0.994 | 1.383 | 0.05912 |
| typehisto_t=Mixte | 0.788 | 2.199 | 1.497 | 3.231 | < 0.001 |
| typehisto_t=Autres | -0.579 | 0.56 | 0.423 | 0.743 | < 0.001 |
| grade_sbr=Grade 2 | 0.313 | 1.368 | 1.19 | 1.571 | < 0.001 |
| grade_sbr=Grade 3 | 0.539 | 1.714 | 1.279 | 2.297 | < 0.001 |
| ss_type2=Luminaux B | 0.235 | 1.265 | 0.916 | 1.746 | 0.15302 |
| ss_type2=Rh+Her2+ | -0.104 | 0.901 | 0.693 | 1.173 | 0.43894 |
| ss_type2=Triple Neg | -0.267 | 0.766 | 0.578 | 1.014 | 0.06215 |
| ss_type2=Rh-Her2+ | 0.345 | 1.412 | 1.017 | 1.961 | 0.03961 |
