# Supplementary material for: Lymph node positivity in different early breast carcinoma phenotypes: a predictive model
Source: BMC Cancer. 2019 Jan 10;19:45. doi: 10.1186/s12885-018-5227-3 (PMC6327612; doi:10.1186/s12885-018-5227-3)

Additional file 2: ***Figure S1*** *Calibration plots of our models.*

*1A: Calibration of predictive LN Involvement for validation set– Pathologic model.*


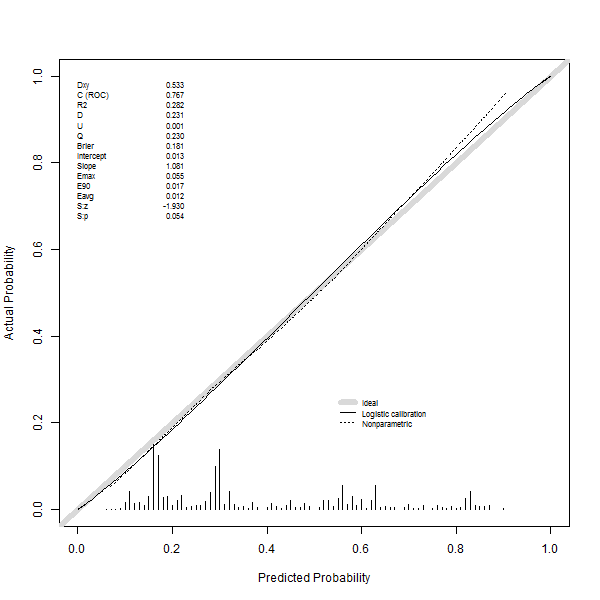


*1B: Calibration of predictive LN macro metastases for validation set – Pathologic model.*


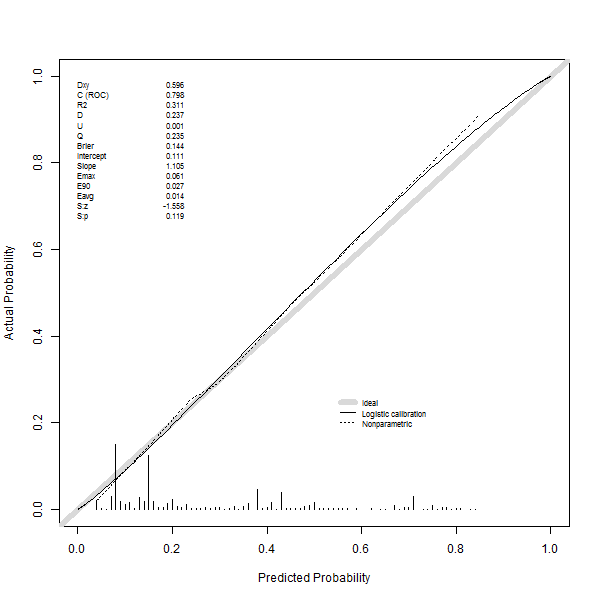


*1C: Calibration of predictive LN Involvement for validation set – Clinical model.*


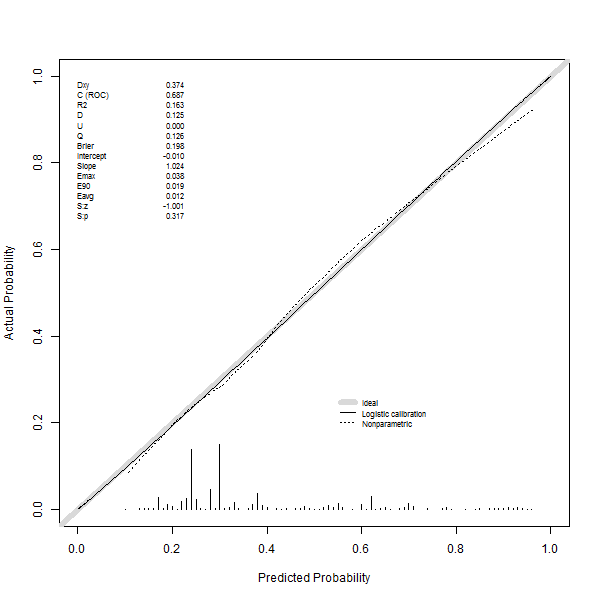


*1D: Calibration of predictive LN macro metastases for validation set – Clinical model.*


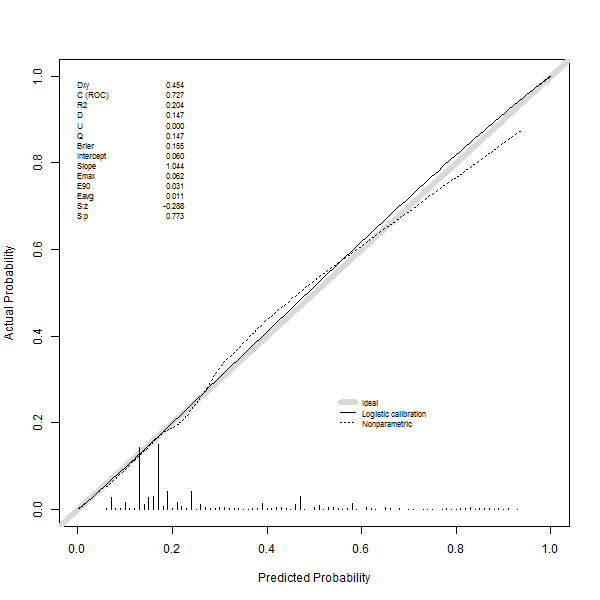

Supplement: Supplementary file 2 — Figure S1. Calibration plots of our models. 1A: Calibration of predictive LN Involvement for validation set– Pathologic model. 1B: Calibration of predictive LN macro metastases for validation set – Pathologic model. 1C: Calibration of predictive LN Involvement for validation set – Clinical model. 1D: Calibration of predictive LN macro metastases for validation set – Clinical model. (DOCX 45 kb) [file 12885_2018_5227_MOESM2_ESM.docx]
