# Supplementary material for: Lymph node positivity in different early breast carcinoma phenotypes: a predictive model
Source: BMC Cancer. 2019 Jan 10;19:45. doi: 10.1186/s12885-018-5227-3 (PMC6327612; doi:10.1186/s12885-018-5227-3)

Additional file 3: ***Figure S2*** *ROC curves of our models.*

*2A: ROC curves of predictive LN Involvement – Pathologic model.*


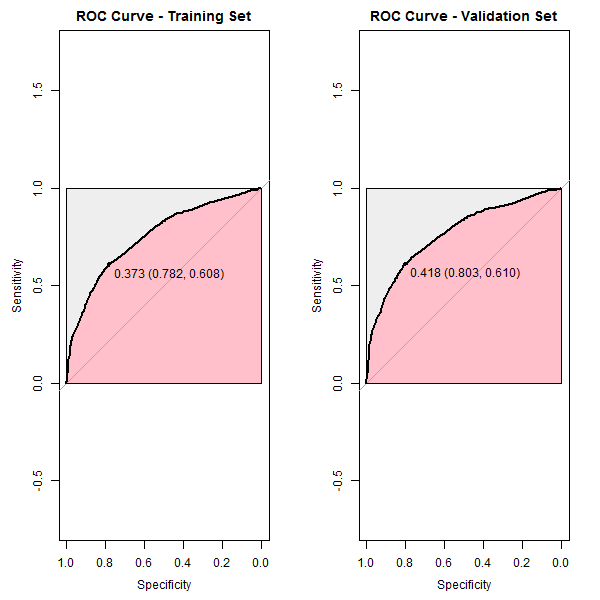


*2B: ROC curves of predictive LN macro metastases – Pathologic model.*


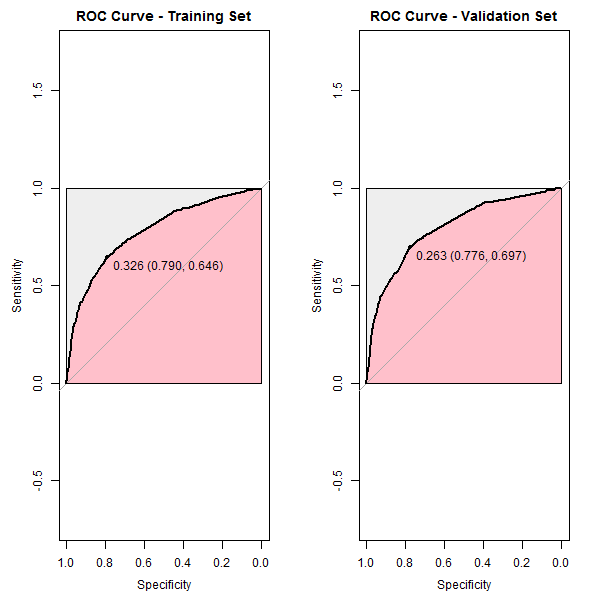


*2C: ROC curves of predictive LN Involvement – Clinical model.*


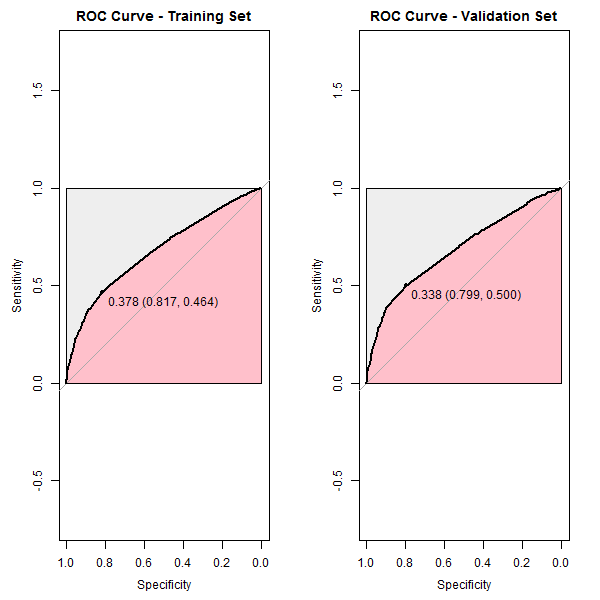


*2D: ROC curves of predictive LN macro metastases – Clinical model.*


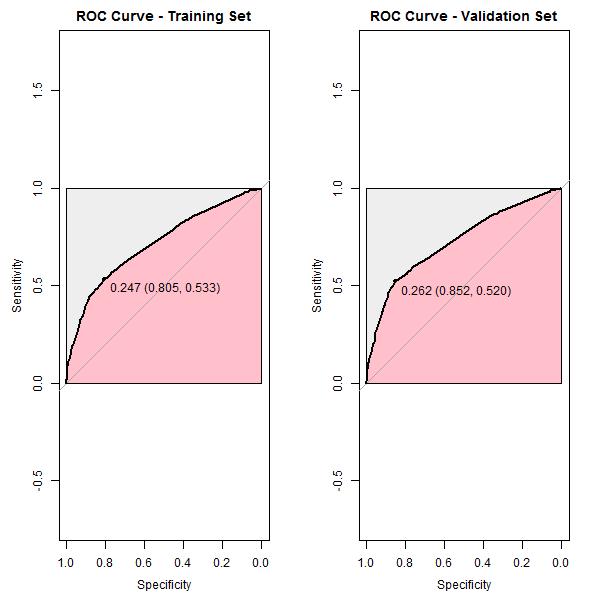

Supplement: Supplementary file 3 — Figure S2. ROC curves of our models. 2A: ROC curves of predictive LN Involvement – Pathologic model. 2B: ROC curves of predictive LN macro metastases – Pathologic model. 2C: ROC curves of predictive LN Involvement – Clinical model. 2D: ROC curves of predictive LN macro metastases – Clinical model. (DOCX 44 kb) [file 12885_2018_5227_MOESM3_ESM.docx]
